# Matrix Information Geometry for Signal Detection via Hybrid MPI/OpenMP

**DOI:** 10.3390/e21121184

**Published:** 2019-11-30

**Authors:** Sheng Feng, Xiaoqiang Hua, Yongxian Wang, Qiang Lan, Xiaoqian Zhu

**Affiliations:** 1 College of Computer Science, National University of Defense Technology, Changsha 410073, China; fengsh14@lzu.edu.cn; 2 College of Meteorology & Oceanography, National University of Defense Technology, Changsha 410073, China; wang_yongxian@hotmail.com (Y.W.); lanqiang_nudt@163.com (Q.L.); zhu_xiaoqian@nudt.edu.cn (X.Z.)

**Keywords:** hybrid MPI/OpenMP, matrix information geometry, parallel optimization, signal detection

## Abstract

The matrix information geometric signal detection (MIGSD) method has achieved satisfactory performance in many contexts of signal processing. However, this method involves many matrix exponential, logarithmic, and inverse operations, which result in high computational cost and limits in analyzing the detection performance in the case of a high-dimensional matrix. To address these problems, in this paper, a high-performance computing (HPC)-based MIGSD method is proposed, which is implemented using the hybrid message passing interface (MPI) and open multiple processing (OpenMP) techniques. Specifically, the clutter data are first modeled as a Hermitian positive-definite (HPD) matrix and mapped into a high-dimensional space, which constitutes a complex Riemannian manifold. Then, the task of computing the Riemannian distance on the manifold between the sample data and the geometric mean of these HPD matrices is assigned to each MPI process or OpenMP thread. Finally, via comparison with a threshold, the signal is identified and the detection probability is calculated. Using this approach, we analyzed the effect of the matrix dimension on the detection performance. The experimental results demonstrate the following: (1) parallel computing can effectively optimize the MIGSD method, which substantially improves the practicability of the algorithm; and (2) the method achieves superior detection performance under a higher dimensional HPD matrix.

## 1. Introduction

Signal detection under a low signal-to-noise ratio (SNR) and complex clutter is a highly challenging task, which is extremely important in signal processing [1]. Due to the presence of complex clutter data, radar target echoes are usually weak and complex, thereby resulting in the failure of the detection performance to meet the application requirements [2]. A classical fast Fourier transform (FFT)-based constant false alarm rate (CFAR) detector is available for addressing this issue. However, this method suffers from severe performance degradation due to the poor resolution and leakage of the spectral energy, thereby resulting in an urgent need for new theoretical support to realize a breakthrough.

**Figure 1:**
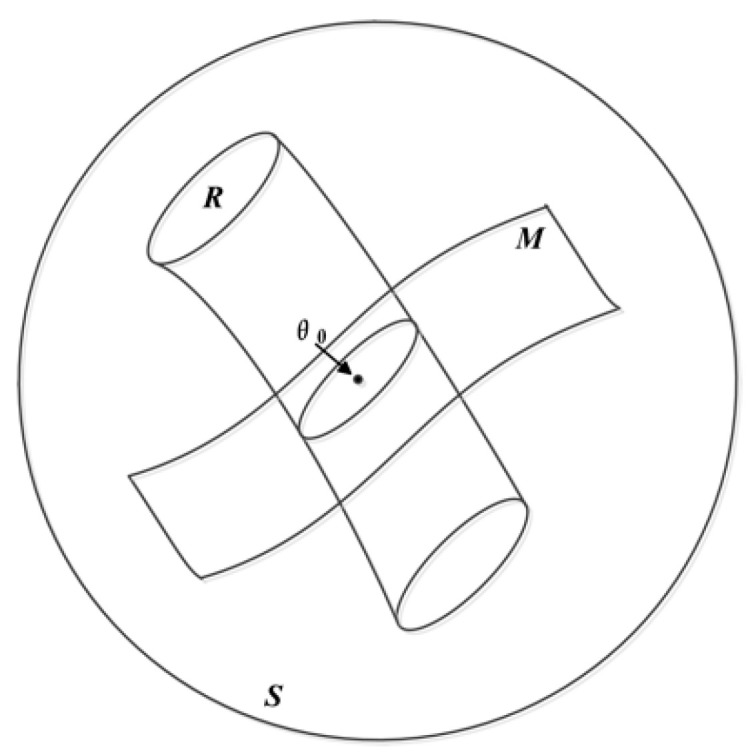
Information geometry-based statistical hypothesis problem.

Information geometry, which is a theory that is based on statistical manifolds, is a differential geometry method for information science problems, which has been applied in numerous areas, e.g., neural networks [3], image processing [4,5,6], information geometric detection [7,8,9,10,11,12], dictionary learning, and sparse coding [13]. Signal detection based on information geometry was first proposed in 1989, when an issue of multisource statistical inference was analyzed and the hypothesis testing problem was explained using a statistical manifold [14], which highlighted the fundamental role that manifold theory plays in statistical information. After that, series of statistical inference theories were investigated via information geometry [15,16]. From the perspective of information geometry, {P(X|θ)} is regarded as a point on a statistical manifold, with a cylindrical confidence zone *R* that is centered on *θ*_0_, where the parameter *θ*_0_ represents the null hypothesis sample data and *M* denotes the statistical manifold. After the statistical modeling from the observed sample data, we can determine whether *θ* is equal to *θ*_0_ or not. Figure 1 illustrates the basic principle of the statistical hypothesis problem.

This novel method, which is based on a Riemannian manifold, provides a new approach for solving complex signal processing problems. In recent years, Barbaresco proposed a CFAR detector that is based on Cartan’s geometry of the Hermitian positive-definite (HPD) product manifold [17,18,19]. The CFAR detector obtains the maximum detection probability while keeping the target detection false alarm rate constant [17], which has become a seminal result in target detection. Furthermore, it is now well established by several studies that the CFAR detector has a large performance advantage in signal processing [9,20]. However, a potential drawback is that the algorithm contains many matrix exponential, logarithmic, and inverse operations, which strongly impact the computational efficiency, as detailed in Section 2. Thus, it is imperative to find an efficient method for optimizing the CFAR detector algorithm, which is also known as the matrix information geometric signal detection (MIGSD) algorithm.

Recently, additional studies and applications in combination with high-performance computing (HPC) methods have been conducted. The practicality of HPC has also been proved, especially for marine and atmospheric numerical calculations. The message passing interface (MPI) began to be widely used in the parallelization of the semi-Lagrangian shallow-water model [21], the parallel ocean model (POM) [22], and the finite-volume coastal ocean circulation model (FVCOM) [23]. The open multiple processing (OpenMP) [24] is extensively applied in the coastal ocean circulation model [25], the mesoscale numerical weather prediction model 5 (MM5) [24], and many other weather forecast models, wave models, and ocean models [26]. In addition, the application of the HPC parallel methods continues to deepen, no longer limited to marine meteorology, but also shines in many other scientific areas, e.g., large-scale image data processing and pattern recognition [27], molecular dynamics [28], computational fluid dynamics applications [29,30], and cosmic celestial motion simulation [31].

In the HPC area, OpenMP realizes superior parallel performance in shared storage environments [32]. MPI is the standard for parallel programming in distributed storage architecture computers [33]. However, due to the rapid growth in the communications between nodes, the bandwidth limits its efficiency; in this case, the use of a single available parallel technology (e.g., MPI or OpenMP) does not yield the desired performance [34,35]. Therefore, we must provide an ideal parallel programming scheme that enables applications to use this hybrid hardware structure most efficiently with minimal overhead and higher performance simultaneously. Fortunately, the hybrid MPI/OpenMP programming model can not only realize two levels of parallelism between nodes but also fully utilize the message passing model and shared-memory programming. The basic strategy of the hybrid MPI/OpenMP programming model is to apply multiple MPI processes on each node with OpenMP threads executing in the MPI process [36,37], which can significantly improve the efficiency of the program.

This hybrid MPI/OpenMP parallel method has been applied extensively for scientific computation. By combining the hybrid MPI/OpenMP modeling with the weather research and forecasting (WRF) model, improved performance over pure MPI or OpenMP has been realized [38]. Duan Geng [39] used the hybrid MPI/OpenMP programming model to improve the KMP algorithm. Phu Luong [40] applied dual-Level Parallelism in coastal ocean circulation modeling. Furthermore, the hybrid parallel method is applied in many new developing fields, e.g., machine learning [41,42,43], data mining [44], and cloud computing [45]. Doubtlessly, hybrid MPI/OpenMP modeling is a classical and promising candidate for scientific application.

The remainder of this paper is organized as follows: First, we introduce information geometry and related information to the MIGSD method. Then, we analyze critical computational components and the computational complexity of the serial algorithm. In Section 3, we present our high-performance computing (HPC)-based MIGSD algorithm, which uses hybrid OpenMP/MPI, and detail our efforts to realize high computational and parallel efficiency on the Tianhe-2 supercomputer. Our experimental results are presented in Section 4 and our ongoing works to overcome the limitations of the current implementations are discussed in Section 5, followed by the conclusions of this study.

## 2. The Matrix Information Geometric Signal Detection Method

In this section, we describe how to map the sample data to a high-dimensional manifold in detail. Then, we derive the Riemannian mean matrix. Finally, we analyze the computational complexity of the MIGSD algorithm. We mention that the manifold is the extension of the concept of curve and surface in high dimensional space, and, if a Riemannian metric can be established in the (local) space of a manifold, the manifold is a Riemannian manifold.

### 2.1. Mapping from the Sample Data to an HPD Manifold

**Figure 2:**
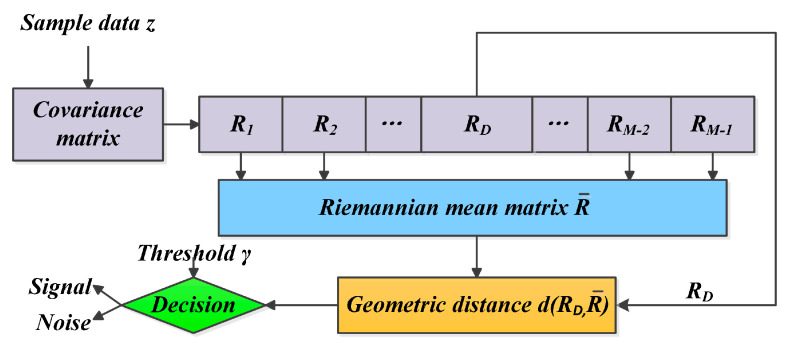
Geometric distance-based the matrix information geometric signal detection method [12].

The main strategy of the MIGSD method is illustrated in Figure 2. The sample data obey the zero-mean complex Gaussian distribution. Since the mean is zero, the information between the sample data is included in the covariance matrix, to which all corresponding distance cells constitute a nonlinear HPD manifold. As an extension of the statistical hypothesis problem, by comparing the geometric distance between the unit matrix and the Riemannian mean matrix with a specified threshold γ, we can judge whether the test cell corresponds to a signal or noise.

For received sample data $z=\{z_{1},z_{2},z_{3},\cdots ,z_{n}\}$, where *n* is the length of the pulse data, the matrix information geometric detector distinguishes the signal from the clutter. Assume that z satisfies a zero-mean complex Gaussian distribution, namely, z~CN(0,H), with the probability density function expressed as follows:

(1) $$p(z;0,H)=\frac{1}{\pi^{n}\left|H\right|}\exp\{-z^{H}H^{-1}z\},$$

where |H| represents the determinant of the covariance matrix, and the covariance matrix ***H*** is formulated as:

(2) $$\begin{array}{l} H=E\left[zz^{H}\right]=\left[\begin{array}{l} h_{0}\;{\bar{h}}_{1}\;\cdots \;{\bar{h}}_{n-1}\\ h_{1}\;h_{0}\;\cdots \;h_{n-2}\;\\ \vdots \\ h_{n-1}\;\cdots \;h_{1}\;h_{0} \end{array}\right],h_{k}=E\left[z_{i}z_{i+k}\right]\\ 0\leq k\leq n-1,1\leq i\leq n \end{array}$$

in which the parameter *h_k_* represents the correlation coefficients, where $\bar{z}$ is the complex conjugate of *z*. ***H*** is essentially a Toeplitz HPD matrix. According to the ergodicity of the stationary Gaussian process, we can calculate *h_k_* by replacing statistical expectations with its time average:

(3) $${\hat{h}}_{k}=\frac{1}{n}\sum_{n=0}^{n-1-\left|k\right|}z(n)\bar{z}(n+k),\left|k\right|\leq n-1,$$

As alluded to above, all the covariance matrices ***H*** corresponding to the distance cells constitute a matrix manifold, which contains the correlation information between the sample data. Thus, the *n*-dimensional vector of the sample data is mapped into an *n*-dimensional matrix space, which can be formed as:

(4) Ψ:ℙ(n)→ℍ(n),z→R∈ℍ(n),

where ℍ(n) represents a Riemannian manifold with nonpositive curvature and ℙ(n) represents the *n*-dimensional vector space, respectively.

### 2.2. Derivation of the Riemannian Mean Matrix

Now, we are ready to derive the Riemannian mean matrix. A manifold ℳ contains a set of points endowed with a curve structure and ***H_i_*** represents an HPD matrix on the manifold ℳ. Between two points ***H*_1_** and ***H*_2_** on ${ℳ}_{ℋ}$, there are infinitely many paths of minimal geodesic distance. In this paper, we measure the distance metric between ***H*_1_** and ***H*_2_** by the geodesic distance, which is formulated as:

(5) $$d_{R}^{2}(H_{1},H_{2})={\left\|\log(H_{1}{}^{-1/2}H_{2}H_{1}{}^{-1/2})\right\|}_{F}^{2}=\sum_{i=1}^{n}\log^{2}(\lambda_{i}),$$

where log(⋅) is the logarithm map on the Riemannian manifold, ‖∙‖_F_ is the Frobenius norm and *λ_i_* represents the *i*-th eigenvalue of $H_{1}{}^{-1/2}H_{2}H_{1}{}^{-1/2}$. The objective function that is used to calculate the mean of data *x* in the Euclidean space is:

(6) $$\bar{x}=\frac{1}{n}\sum_{i=1}^{n}x_{i}=\underset{x>0}{\arg\min}\frac{1}{n}\sum_{i=1}^{n}\left|x-x_{i}\right|.$$

For the HPD manifold, we employ the geometric distance instead of the Euclidean distance. Let $\left\{H_{1},H_{2},H_{3},\cdots ,H_{n}\right\}$ denote a set of HPD matrices, with the mean matrix $\bar{H}$ defined as follows.

(7) $$\bar{H}=\underset{H}{\arg\min}\frac{1}{n}\sum_{i=1}^{n}d(H,H_{i}).$$

The subgradient algorithm is used to run the iteration via the fixed-point method [46,47]. Its convergence can be proved as follows: For a set of HPD matrices, the objective function *F*(***H***) can be expressed as:

(8) $$F(H)=\frac{1}{n}\sum_{i=1}^{n}d^{2}(H,H_{i})=\frac{1}{n}{\sum_{i=1}^{n}\left\|\log(H^{-1}H_{i})\right\|}_{F}^{2}.$$

Then, the gradient ∇F is derived.

(9) $$\nabla F=\frac{1}{n}\sum_{i=1}^{n}2\log(H_{i}^{-1}H)H^{-1}.$$

Let ∇F=0, ***H*** is a positive matrix. Thus,

(10) $$\sum_{i=1}^{n}\log(H_{i}^{-1}H)=0.$$

For these *n* HPD matrices $\left\{H_{1},H_{2},H_{3},\cdots \;\cdots ,H_{n}\right\}$, both sides are multiplied by ***H***_1_^−1/2^, and then we have $\left\{I,H_{1}^{-1/2}H_{2}H_{1}^{-1/2},H_{1}^{-1/2}H_{3}H_{1}^{-1/2},\cdots ,H_{1}^{-1/2}H_{n}H_{1}^{-1/2}\right\}$. In this way, the above sequence can be rewritten as $\left\{P_{1},P_{2},P_{3},\cdots ,P_{n}\right\}$. According to the congruent transformation in Riemannian geometry, these matrices are still on the HPD manifold, and the Riemannian mean matrix does not change. Then,

(11) $$\sum_{i=1}^{n}\log(P_{i}^{-1}P)=0.$$

Since ***P***_1_ represents the unit diagonal matrix ***I***. Thus,

(12) $$\log(P)=-\sum_{i=2}^{n}\log(P^{1/2}P_{i}^{-1}P^{1/2}).$$

For simplicity, let S=log(P), namely, P=exp(S). Then,

(13) $$P^{1/2}=\exp(\frac{1}{2}S).$$

According to the fixed-point method, the iterations can be formulated as follows:

(14) $$\begin{array}{c} S_{0}=\frac{1}{n}\sum_{i=1}^{n}\log(P_{i})\\ S_{t+1}=\alpha S_{t}+(\alpha -1)\sum_{t=2}^{n}\log(\exp(S_{t}/2)P_{i}^{-1}\exp(S_{t}/2))\\ 0<\alpha <1 \end{array}$$

Applying the logarithm map yields:

(15) $$P_{t+1}=P_{t}^{1/2}\exp(\eta \sum_{i=1}^{n}\log(P_{i}^{-1/2}P_{i}P_{i}^{-1/2}))P_{t}^{1/2},$$

where η=1−α denotes the stepsize and *t* the number of the iterations. The above equation converges after many iterations to the Riemann mean. As discussed above, our objective is to calculate the geometric distance between the test cell and the Riemann mean matrix $\bar{P}$; in this case, signal detection is performed via comparison with a threshold.

### 2.3. Computational Complexity of the Algorithm

To set the stage for the algorithm complexity analysis, we recall useful information regarding computational complexity, which is shown in Table 1. Then, we detail the serial MIGSD algorithm based on MATLAB (R2019a) to analyze the computational complexity. Arithmetic with individual elements has complexity *O*(*1*).

*Pd_D* is the signal detection rate. *PFA* denotes the desired probability of false alarm, through which the threshold is determined. The signal-to-noise ratio (SNR) is defined as follows:

(16) $$SNR=10\log_{10}\frac{P_{l}}{\sigma^{2}},$$

where *P_l_* is the signal power received by radar and *σ^2^* represents the noise variance. The higher is the SNR, the smaller is the amount of noise that is mixed with the signal (the higher the signal quality).

Now, we are ready to describe the serial method by presenting the main pseudocode in Algorithm 1, from which it is clear that the MIGSD algorithm involves a double-loop: *Pd_D* is initialized in the outer loop, while the inner loop executes many matrix operations. The calculation task in the inner loop can be divided into three main parts: the estimation of the Toeplitz matrix, the calculation of the Riemannian mean matrix, and the calculation of the geodesic distance.

**Algorithm 1***MIGSD* (*M*, *K*, *PFA*, *Pd_D*)

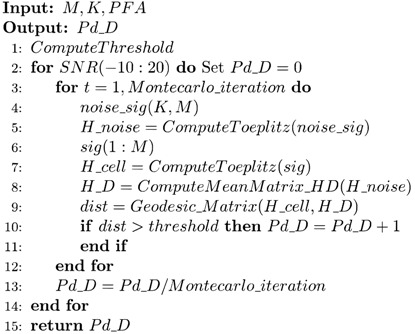



Each test cell is mapped into an HPD matrix on the Riemannian manifold. The *threshold* is determined by numbers of matrix iterations according to *PFA*. To obtain the signal detection rate *Pd_D*, the Monte Carlo method is employed inside the double loop. This method uses a weighted random sample to simulate the posterior probability distribution of the solution, which is also known as a random sampling method, to transform the integral into a summation form. Each Monte Carlo process compares the *Geodesic_Matrix* (*H_Cell*, *H_D*) and the *threshold* to solve the *Pd_D* problem.

The computational complexity of the mean matrix can be upper bounded by counting the number of multiplication operations. In this case, the Riemannian mean can be evaluated via an iterative procedure with (3*an*^3^ + *an*^2^)*tk* multiplications, where *a* denotes the number of HPD matrices for averaging, *tk* is the number of iterations, and *n* is the length of the pulse data in the range cell, which is substantially more expensive. The high computational cost limits its practical application, thereby making it much more difficult to evaluate the detection performance as the dimension increases, which is discussed in Section 4.1.

## 3. High-Performance Computing-Based MIGSD Method

As discussed above, the high computational cost of the MIGSD algorithm poses challenges in analyzing the relationship of the detection performance and the dimension of the HPD matrices, which motivates us to use a high-performance computing (HPC) method to accelerate the algorithm. Moreover, as there are no data correlation in the iterative procedure, the hybrid MPI/OpenMP model can be employed effectively to improve the MIGSD algorithm. However, these methods are not supported in MATLAB. Thus, we transform the MATLAB program into a Fortran90 version to apply the HPC methods. From the perspective of HPC, our primary objective is to identify the hotspots of the program, from which we can obtain the largest performance improvement. The hotspots of the MIGSD serial program are concentrated in the double loops for the solution, for instance, the Monte Carlo iterations for the solution; the iterative method for calculating the mean matrix; and the matrix inverse, logarithm, and eigenvalue operations for calculating both the mean matrix and the geodesic distance.

**Figure 3:**
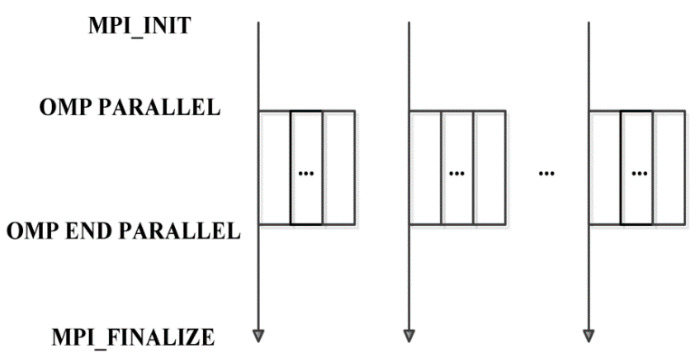
The framework of the hybrid MPI/OpenMP programming model.

Now, we present our parallel algorithm, which is detailed as follows:

The HPC-based MIGSD algorithm is divided into training and working steps: the training step provides the *threshold*, while the signal detection rate *Pd_D* is calculated in the working step. As discussed above, since the distance between every pair of points in the HPD manifold is independent, the current calculated distance does not affect the next calculated distance in the main loop. In this case, MPI can be applied in the outer layer to create processes, while OpenMP is used in the inner layer to create threads. This framework of the hybrid MPI/OpenMP programming model fully utilizes the bandwidth, as illustrated in Figure 3. MPI_INIT is used to initialize the MPI environment to establish links between multiple MPI processes, while OMP PARALLEL opens the multithread environment. MPI_Finalize is used at the end of the MPI runtime environment, while OMP END PARALLEL closes the multithread parallel domain. These functions are the basic parallel framework for defining MPI or OpenMP programs.

### 3.1. Our Efforts in the Training Step

In the training step, our objective is to identify a *threshold* from the sequence of geodesic distances according to the *PFA*. To this end, MPI is used for task partitioning, while OpenMP is fused on the MPI-divided loop for further computation, namely, the task is divided by MPI processes, while OpenMP threads compute the local geodesic distance via involved functions in the MPI processes. After every process has completed the assigned calculation tasks, namely, the OpenMP threads have computed all the local distances, we gather all the local distances into the main process using function MPI_GATHER. In this case, the main process contains the full sequence of geodesic distances, which enables us to sort the geodesic distances into descending order. In addition, the *threshold* is determined in this descending sequence according to the *PFA*. To facilitate comparison with the threshold during the working step, the *threshold* will be broadcasted to the other MPI processes using the function MPI_BCAST. In this way, each MPI process has a copy of the *threshold*. This concludes the training step. Since each computation in the training step is independent with nearly no process communication overhead, the HPC-based MIGSD algorithm realizes high parallel performance.

The pseudocode of the training step in the HPC-based MIGSD program is presented as Algorithm 2. The parameter *myrank* indicates the process number, *npros* means the number of MPI processes, *distlist_descent* represents the descending sequence for *distlist*, and *t* represents the maximum number of training.

**Algorithm 2** Training (*M*, *K*, *PFA*, *threshold*)

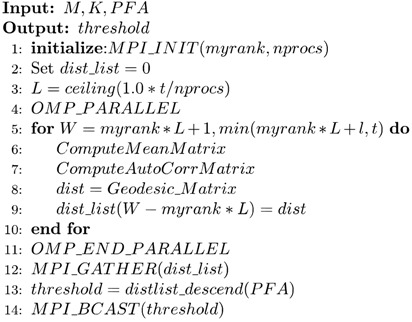



### 3.2. Our Efforts in the Working Step

The working step is similar to the training step. The main difference is that we use the Monte Carlo method for all SNRSs through a double loop. Since the *threshold* that is obtained from the training step has already been passed to each process by function MPI_BCAST, we can immediately employ the Monte Carlo method in each process to compare *dist* and *threshold*. The input data to the working step are a clutter-containing signal matrix that is generated by a random function, and the MPI environment exists until the instruction MPI_FINALIZE is encountered.

In the Monte Carlo iteration, the correlation matrix, the mean matrix, and the geodesic distances of these two matrices are calculated without interference, which is known as task-level parallelism. Moreover, since the operations of each iteration are independent, MPI can be used outside the Monte Carlo loop, namely each MPI process executes a part of the Monte Carlo iterations, while multicore OpenMP is used in the MPI process to calculate the involved functions. In each OpenMP thread, *dist* is compared with *threshold*; in this case, the signal is finally determined, which is denoted as a *cnt*. The parallel domain of OpenMP is closed until all *cnts* have been obtained. At this point, the Monte Carlo simulation is also complete. We turn to outside of the Monte Carlo loop, where MPI_REDUCE is used to sum the signals in the subprocesses. In this case, the signal detection rate, namely *Pd_D*, is obtained from the working step. The basic parallel implementation of the working step is presented in Algorithm 3. The parameter *Num_Montecarlo* means the number of Monte Carlo iterations, *myrank* indicates the process number, *npros* means the number of MPI processes, and *cnt_total* represents the total number of signals obtained after comparison with the *threshold*.

**Algorithm 3** Working (*M*, *K*, *threshold*, *Montecarlo*)

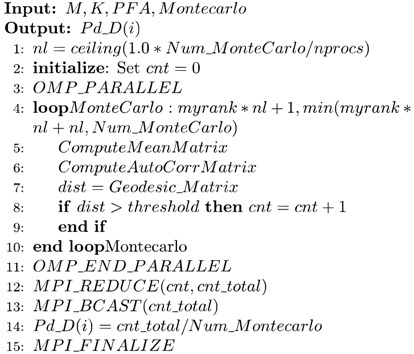



In this application, since the program rarely incurs communication overhead, our HPC-based MIGSD program can realize high parallel optimization performance both within nodes and between nodes. The performance of our hybrid scheme is strong and it remains so for any combination of MPI processes and OpenMP threads, which is detailed in Section 4 (it provides nearly linear speed-up).

## 4. Numerical Experiments

Several experiments were conducted to evaluate both the serial and HPC-based MIGSD programs. We employed three parallel schemes in our experiments: (1) an MPI-only scheme; (2) an OpenMP-only scheme; and (3) a hybrid MPI/OpenMP scheme. We considered the time cost of the serial MIGSD program based on Fortran as the dimension of the HPD matrix increases. Then, we evaluated the parallel performance of our HPC-based MIGSD program. Moreover, we evaluated the detection performance of the higher-dimensional HPD matrix via our hybrid MPI/OpenMP algorithm. Finally, the relationship between the dimension and the detection performance was identified. Table 2 presents the test platform and environment in our experiments.

### 4.1. Time Cost in the Serial MIGSD Program

As discussed above, the computational complexity of the serial MIGSD algorithm is huge due to the matrix operations, the iterations for calculating the mean matrix, and the Monte Carlo method in the inner loop.

**Figure 4:**
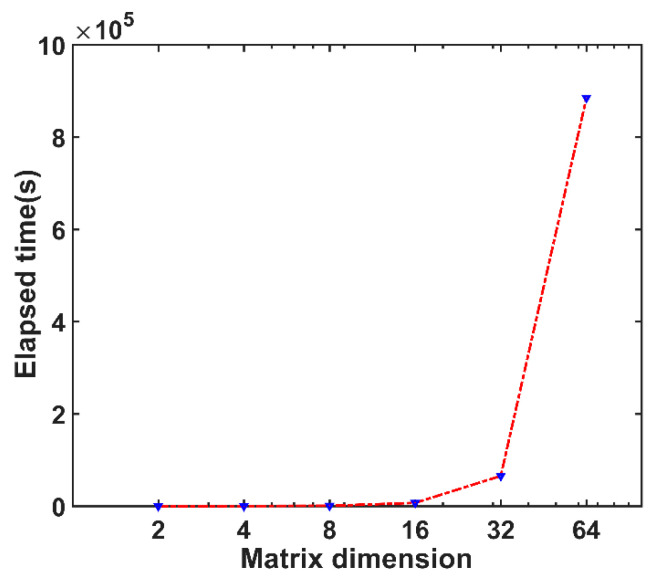
The elapsed time of the serial MIGSD program experiment groups.

We now consider the run time of the Fortran-based serial MIGSD program as the HPD matrix dimension increases, which is plotted in Figure 4. The time cost grows rapidly as the dimension of the matrix increases. More specifically, in the case of low matrix dimension, the program can be optimized by the internal hardware to a certain extent, and the calculation data can be easily stored by the computer. In this case, the time growth is not very fast. However, as the dimension increases, it is impossible to process such a large amount of data internally by the computer, which results in an exponential growth of the time consumption. In particular, when the dimension is set to more than 64, it may run more than 240 h, which is surprisingly enormous. In fact, it is expected to grow faster in higher dimensions. This is further confirmed that the algorithm has high computational complexity, and difficulties are encountered in testing the detection performance for a higher dimensional matrix.

### 4.2. Parallel Performance in HPC-BASED MIGSD Program

To evaluate the parallel performances of the HPC-based MIGSD program, we tested this hybrid MPI/OpenMP program with various numbers of threads and processes. A speed-up metric was used to quantify the parallel performance:

(17) $$S_{p}=T_{s}/T_{p},$$

where *p* represents the number of the processes, *T_S_* is the execution time of the sequential algorithm, and *T_p_* is the execution time of the parallel algorithm with *p* processors, respectively. Each node in the Tianhe-2 supercomputer has 24 cores. Schemes (1) and (2) were both tested in a single node. Thus, we set 1, 2, 4, 8, and 16 processes or threads to analyze the variation trend of the speed-up. Note that the dimension of the HPD matrix in this section was set to 4, 8, and 12, and the *PFA* was set to 10^−3^ to facilitate our tests.

**Figure 5:**
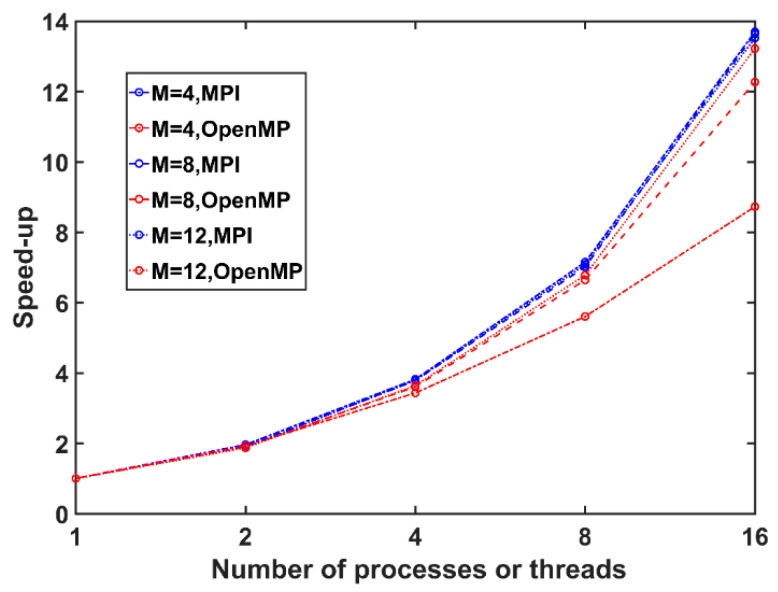
Speed-up comparison between the MPI-only scheme and the OpenMP-only scheme. The parallel performance is quantified by the speed-up metric (as defined above). Different lines represent the results of different matrix dimensions. The blue line represents the results of Scheme (1), while the red line represents the results of Scheme (2).

According to Figure 5, the speed-ups of Schemes (1) and (2) both show a strong upward trend with the MPI and OpenMP techniques. In the beginning, the application of MPI and OpenMP provides nearly linear speed-up, although the parallel efficiency decreases as the number of processes or threads increases, which we attribute to the bottleneck of the problem magnitude. In addition, the two curves are similar with few processes and threads; hence, Schemes (1) and (2) provide a similar parallel performance initially, i.e., the speed-ups of the MIGSD program that are based on MPI or OpenMP are similar under the same numbers of processes and threads. With the increase in the numbers of processes and threads, Scheme (1) outperforms Scheme (2), i.e., MPI processes provide more parallel efficiency than OpenMP threads, together with more advantages within nodes. A possible reason is that the MPI model is employed outside the loop, basically throughout the whole program, that is, the MPI environment is opened before the OpenMP environment. In this case, the MPI process divides tasks before the OpenMP thread, resulting in more parallelism achieved by the MPI model in our algorithm. In general, this phenomenon is more obvious in the case of a low dimension. As the dimension increases, the parallel performance achieved by MPI is basically the same, while OpenMP achieves higher performance in higher dimensions, although it is still worse than MPI. Moreover, the MPI programming model inherently imposes better data locality than OpenMP.

An additional experiment was conducted in Scheme (3) to evaluate the within-node parallel performance of the hybrid MPI/OpenMP program. More specifically, the dimension was set to 8 in this experiment. The number of MPI processes times the number of OpenMP threads was fixed to 24 to fully utilize the 24 cores in one Tianhe-2 node: (a) 1 thread, 24 processes; (b) 2 threads, 12 processes; (c) 3 threads, 8 processes; (d) 4 threads, 6 processes; (e) 6 threads, 4 processes; (f) 8 threads, 3 processes; (g) 12 threads, 2 processes; and (h) 24 threads, 1 process. We compared the parallel performance of these eight experimental groups. According to Table 3, Combinations (g) and (h) result in significantly slower elapsed times and lower speed-up, while the other combinations have approximately the same parallel performance. Hence, the combinations of MPI processes and OpenMP threads may influence the parallel performance.

**Figure 6:**
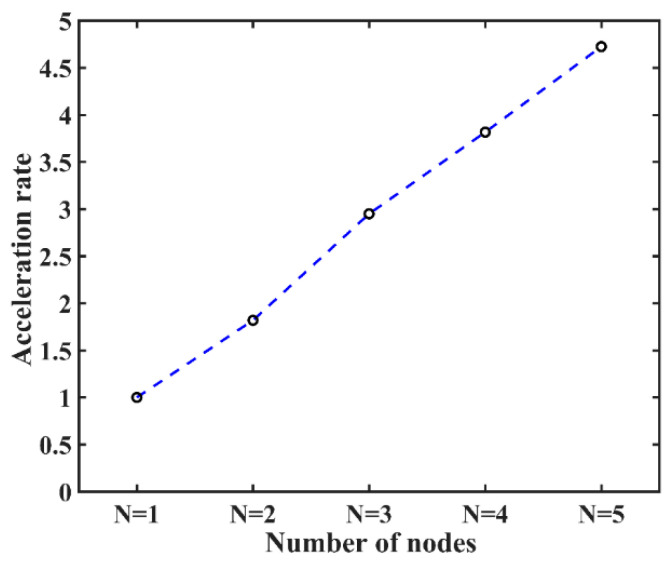
Parallel performances of multi-nodes. The *x*-axis represents five experimental groups under different number of nodes, where *N* represents the number of nodes. The *y*-axis represents the acceleration rate.

In the scalability experiment, we attempted to identify additional parallelism in multinodes to increase the parallel efficiency. The size of the problem and the number of OpenMP threads (export OMP_NUM_THREADS = 24) were fixed, while the numbers of nodes and MPI processes were changed from 1 to 5 to obtain five experimental groups. N represents the number of nodes, n is the number of MPI processes, and the 24 cores of a single node in the Tianhe-2 supercomputer were fully loaded. The acceleration rate denotes the ratio of the running time under one single node to the time under multiple nodes. As illustrated in Figure 6, the result demonstrates that the multinode parallelism in our hybrid MPI/OpenMP program has high scalability: the acceleration rate compared to a single node maintains approximately linear growth as the number of nodes increases; hence, our HPC-based MIGSD program can also realize high parallel performance in the case of multinodes. It is possible that the additional overhead of between-node communication of our HPC-based MIGSD program is small. In this case, it becomes much easier to evaluate the detection performance of the high-dimensional HPD matrix.

### 4.3. Detection Performances for Various Dimensions of the Matrix

Facilitated by hybrid MPI/OpenMP parallel modeling, our approach leads to a significant improvement gain over the MATLAB version. In this case, the detection performance can be tested with high efficiency. In our experiment, the dimension of the HPD matrix M ranged from 8 to 72 with an interval of 8. The number of sample data was *K* = *2M*, the SNR varied from −10 to 20 dB with an interval of 1 dB, and the multinode parallelism of our HPC-based MIGSD algorithm was employed for this test.

**Figure 7:**
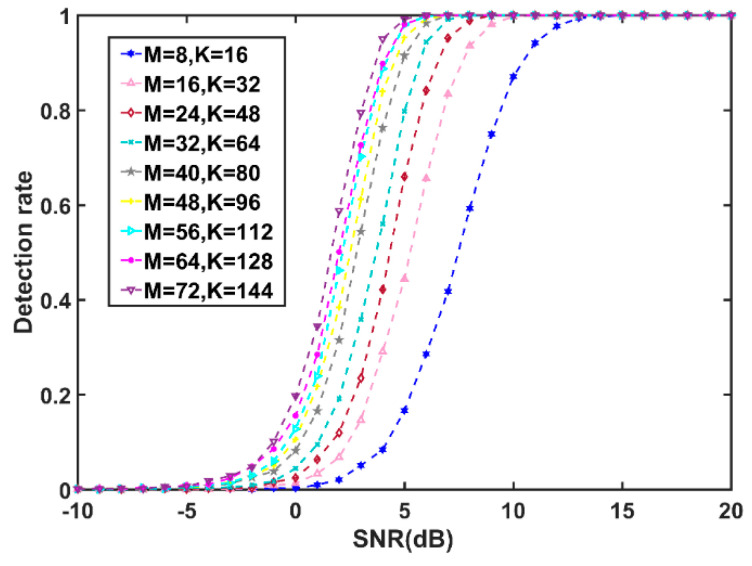
Detection probability curves for various matrix dimensions. The number of dimensions ranged from 8 to 72 with an interval of 8. The SNR denotes the signal-to-noise ratio, which varied from −10 to 20 dB with an interval of 1 dB.

According to Figure 7, the detection performance increases with the matrix dimension, that is the effect of clutter on detection performance is becoming less and less, although the increase slows at high dimensions. For a high-dimensional HPD matrix, the signal detection performance can be improved at low SNR. When SNR is 5, the detection performance of the case that *M* = 48 has almost reached 1, while the detection probability is very low in the case *M* = 8, the detection performance difference between the two is more than 5 dB. It is possible that sufficient information on the clutter or the target signal can be provided by the high-dimensional covariance matrix, which makes the algorithm achieve better detection performance.

## 5. Discussion

The influence of the noise that is mixed into the signal on signal processing is a subject that merits further study. However, due to the variety of clutter and the large amount of data, accelerating the detection and processing of radar signals is a difficult problem. In this paper, we use the hybrid MPI/OpenMP parallel model to overcome the high complexity and the large computational cost of the MIGSD method. In addition, the detection performance of the MIGSD method with a variety of dimensions is explored, which is especially important for practical applications.

The experimental results clearly demonstrate the following: (1) Parallel tools can accelerate the MIGSD algorithm, and, interestingly, computer technology and signal detection are fused. (2) The detection performance of the MIGSD algorithm varies with the dimension of the HPD matrix. The higher the HPD matrix dimension is, the better the detection performance of the matrix information geometric detector is.

For future research, we may focus on selecting a suitable *PFA* in the MIGSD method and on using other HPC methods (e.g., acceleration methods that are based on GPU hardware) to complete our HPC-based MIGSD program. Additionally, since the high memory usage becomes a substantial problem as the dimension of the HPD matrix increases, we consider optimizing the serial MIGSD algorithm; perhaps other Riemannian distance metrics are suitable.

## Figures and Tables

**Table 1:** Computational complexity of various vector and matrix operations.

Operation	Complexity
Vector addition	*O*(*n*)
Vector multiplication	*O*(*n*^2^)
Matrix addition	*O*(*n*^2^)
Matrix multiplication	*O*(*n*^3^)
Matrix eigenvalue	*O*(*n*^3^)
Matrix logarithm	*O*(*n*^3^)
Matrix inversion	*O*(*n*^3^)

**Table 2:** Test platform and environment.

Item	Values
2 × CPU	Intel(R) Xeon(R) CPU E5-2692 v2 @ 2.20 GHz
Operating System	Kylin Linux
Kernel	2.6.32-279-TH2
MPI Version	MPICH Version 3.1.3
GCC Version	GCC 4.4.7
Compiler	Intel-compilers/15.0.1

**Table 3:** Parallel performance of various combinations of MPI processes and OpenMP threads in one single node.

Experimental Groups	Elapsed Time (s)	Speed-Up
(a) 1 thread, 24 processes	39.49	**20.44**
(b) 2 thread, 12 processes	41.61	19.40
(c) 3 threads, 8 processes	41.3	19.54
(d) 4 threads, 6 processes	41.50	19.54
(e) 6 threads, 4 processes	42.46	19.01
(f) 8 threads, 3 processes	42.53	19.00
(g) 12 threads, 2 processes	80.7	10.01
(h) 24 threads, 1 process	51.53	15.67
